# Microstructure and Tribological Properties of Mo–40Ni–13Si Multiphase Intermetallic Alloy

**DOI:** 10.3390/ma9120986

**Published:** 2016-12-06

**Authors:** Chunyan Song, Shuhuan Wang, Yongliang Gui, Zihao Cheng, Guolong Ni

**Affiliations:** School of Metallurgy and Energy, North China University of Science and Technology, Tangshan 063009, China; scy@ncst.edu.cn (C.S.); wshh88@ncst.edu.cn (S.W.); 18232532877@163.com (Z.C.); 15032913913@163.com (G.N.)

**Keywords:** intermetallic, refractory metal, tribological property, toughening

## Abstract

Intermetallic compounds are increasingly being expected to be utilized in tribological environments, but to date their implementation is hindered by insufficient ductility at low and medium temperatures. This paper presents a novel multiphase intermetallic alloy with the chemical composition of Mo–40Ni–13Si (at %). Microstructure characterization reveals that a certain amount of ductile Mo phases formed during the solidification process of a ternary Mo–Ni–Si molten alloy, which is beneficial to the improvement of ductility of intermetallic alloys. Tribological properties of the designed alloy—including wear resistance, friction coefficient, and metallic tribological compatibility—were evaluated under dry sliding wear test conditions at room temperature. Results suggest that the multiphase alloy possesses an excellent tribological property, which is attributed to unique microstructural features and thereby a good combination in hardness and ductility. The corresponding wear mechanism is explained by observing the worn surface, subsurface, and wear debris of the alloy, which was found to be soft abrasive wear.

## 1. Introduction

Wear, one of the most frequently encountered failure models for engineering materials, leads to huge economic losses every year. There has been significant interest among the members of the scientific community in developing a variety of wear resistant materials [1,2]. Traditional metallic engineering materials designed for structural application find it difficult to satisfy the requirements of higher and higher operating stresses and temperatures in mechanical moving components. Intermetallic compounds are attracting growing attention due to their inherent strong atomic bonds and high hardness, promising good wear resistant materials [3,4,5,6].

Over the past couple of decades, binary intermetallic compound NiMo with a topologically close-packed δ-phase structure, was studied widely because of its attractive thermodynamic properties [7,8,9]. However, little attention was given to NiMo as a wear material or as a coating, even though some made efforts on its mechanical properties [10]. From the tribological point of view, the covalent-dominant strong atomic bond endows intermetallic NiMo outstanding adhesive wear resistance, and the high hardness and anomalous hardness-temperature relation provide an excellent abrasive wear resistant property [11]. Unfortunately, monolithic NiMo is extremely brittle for structural application. Hence, how to improve the toughness of NiMo at low and medium temperature, like other intermetallic compounds, becomes a great and continuous challenge.

To improve room-temperature ductility of intermetallic compounds, recent trends have been to make multiphase composites by introducing a second and even a third phase which neighbors in the equilibrium phase diagram [12]. The research of Liu et al. discovered that the Nb_SS_ phase has a remarkable toughening effect in an alloy consisting of Nb_SS_ and Nb- and Ti-rich silicides, and observed slip steps inside the Nb_SS_ during crack propagation, which indicated deformation of the alloy occurring in the Nb_SS_ [13].

A great number of works have proven molybdenum, as well as other refractory metals, possesses excellent ductility, toughness, and a high melting point, implying it is an ideal toughening phase for the intermetallic compound [14,15,16,17,18]. For instance, the alloy consisting of Mo_3_Si and Mo_5_SiB_2_ (T2) as well as Mo-based solid solution phase has shown satisfactory higher fracture toughness values than monolithic Mo_3_Si of T2 [19]. Additionally, for the introductory approach, in situ incorporation of ductile refractory metals into intermetallic compounds has been demonstrated to be effective and practical [20,21,22]. An α-Mo phase, with bcc crystal structure, has been in situ formed successfully in several molybdenum-based metallic silicide alloys as a strategy for improvement of ductility and toughness [23,24,25]. In the light of above understanding, the method of in situ incorporation was employed and the ductile metallic Mo was selected as the toughening phase for the intermetallic compound NiMo in the present investigation. Regarding wear resistance, intermetallic composites toughened by ductile metals could be better than non-toughened, even though it may somewhat offset some deleterious effects associated with the decrease in hardness [26].

Another focus in this novel alloy design is on understanding that the in situ formation of metallic silicides in Mo–Ni–Si system may further optimize the properties of intermetallic alloy, such as good creep resistance, low density, and wear resistance. Therefore, additional Si was added intentionally in the chemical composition for the purpose of achieving one or more in situ metallic silicide phases. To be satisfied, a certain amount of Mo_2_Ni_3_Si phase was identified in novel Mo–40Ni–13Si alloy through the appropriate design of chemical composition and careful control of the manufacturing process. It is better that Mo_2_Ni_3_Si ternary metal silicide formed in the solidification process than appearance of binary metal silicides, because ternary metal silicides exhibit better mechanical properties resulting from the comparatively weaker atomic bonding [27]. Furthermore, metallic slilicide Mo_2_Ni_3_Si has relatively good toughness and high hardness, and is a promising reinforcement phase for wear resistant metallic materials [28].

In this work, we proposed a novel multiphase intermetallic alloy in ternary Mo–Ni–Si system which is designed to be used as a structural coating material in wear environments. Microstructure was characterized and solidification process of novel intremetallic alloy was analyzed. Tribological properties were evaluated under dry sliding wear test conditions at room temperature, and the governing wear mechanisms were discussed according to the examination of the worn surface, debris, and subsurface with scanning electron microscopes (SEM). To rank the improvement of wear resistance, the potential matrix steel materials, hardened 0.45%C steel and austenitic 1Cr18Ni9Ti stainless steel, were selected as wear test reference materials.

## 2. Experimental Procedures

### 2.1. Alloy Preparation

The Mo–40Ni–13Si alloy was manufactured using commercially pure molybdenum (99.9%), nickel (99.5%), and silicon (99.96%) with a particle size of 40 to 74 μm. The proportional Mo–Ni–Si powder blends in the chemical composition Mo–40Ni–13Si (at %)were preheated at 120 °C for 4 h to thoroughly eliminate the absorbed moisture, and then melted using an arc-melting furnace in argon atmosphere. The technical parameters for the fabricating process were taken according to preliminary works, which are optimized to be an electric current of 300 A, voltage of 10–12 V, and the pressure of 65 kPa. All ingots were remelted three times to make it homogeneous prior to use for microstructure characterization, hardness, and wear testing.

### 2.2. Microstructural Characterizations and Hardness Tests

Several typical ingots of Mo–40Ni–13Si alloy were cut along the vertical section in the middle for microstructure characterization. The metallographic samples were prepared using standard mechanical polishing (ground using a range of 400–1500 grinding paper and polished by diamond paste from 6 to 1 μm) and chemical etching procedures (etched in HF:HNO_3_:H_2_O solution with volume ratio of 1:6:7). The observations of microstructure were carried out using Axiovert 200mat invert-type optical microscope (OM, Carl Zeiss Light Microscope GmbH, Göttingen, Germany) and KYKY-2800B scanning electron microscope (SEM, KYKY Technology Development Ltd., Beijing, China) with secondary electron imaging mode and energy dispersive spectroscopy (EDS, KYKY Technology Development Ltd., Beijing, China) analysis. X-ray diffraction (XRD, Rigaku Corporation, Tokyo, Japan) analyses were conducted to verify the phase constituents of the alloy by D/MSX2500PC X-ray diffractometer using Cu Kα radiation with a scanning rate of 5°/min.

The micro-hardness of Mo–40Ni–13Si alloy was measured under a load of 500 g and a load dwell time of 15 s using a digital HXZ-1000 micro-hardness indenter (Shanghai Optical Instrument Factory, Shanghai, China). The micro-hardness value of this alloy was determined by taking the mean of at least five successful measurements. For quantitative analysis of the microstructure, linear intercept method was used for the purpose of determining the volume fraction of individual phase.

### 2.3. Wear Tests

All block-like Mo–40Ni–13Si alloy specimens for wear tests were extracted from the central regions of the arc-melted ingots in the cubic form with a size of 10 mm × 10 mm × 10 mm. The highest potential application of the Mo–40Ni–13Si alloy is as a coating material on engineering steel matrixes. Hence, two most-widely applied engineering materials, hardened 0.45%C steel and austenitic 1Cr18Ni9Ti stainless steel, were selected as comparison test materials in order to rank the increase in wear resistance. The hardnesses of reference hardened 0.45%C steel and austenitic stainless steel 1Cr18Ni9Ti are about HV260 and HV640, respectively.

Prior to wear tests, sample preparation involving grinding and polishing were performed with optimum procedures to achieve repeatable results, considering the effect of roughness and flatness on wear process, especially in the initial stage. The surfaces of the samples were ground using 600, 800, 1200, and 1500 grinding papers and then polished with 6, 3, and 1 μm diamond pastes, after which the samples were cleaned with acetone in ultrasonic cleaner.

**Figure 1:**
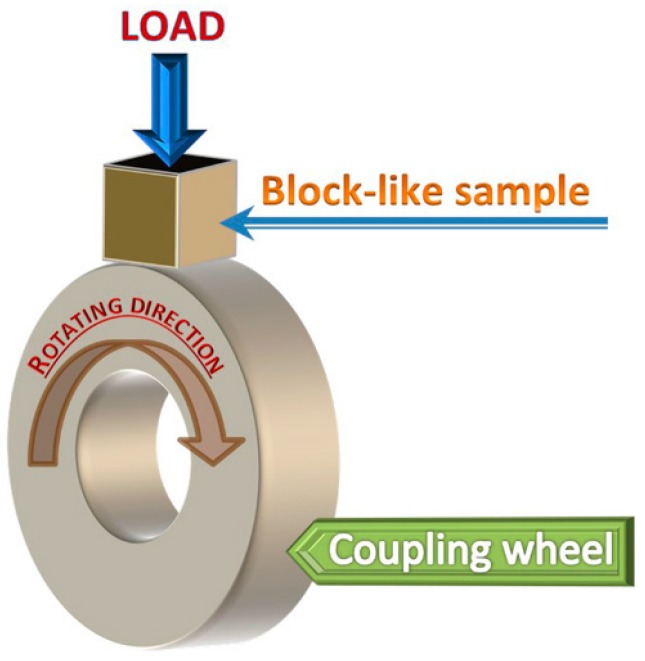
Schematic illustration of block-on-wheel mode dry sliding wear test.

The sliding friction and wear tests were carried on a MM-200 type block-on-wheel mode machine, the schematic diagram of which is given in Figure 1. The block-like specimen is pressed against the outer periphery surface of a hardened 1.0%C–1.5%Cr bearing steel wheel (measured hardness of HRC63 ± 1) rotating at 400 rpm. Wear tests were conducted at a 49, 98, 147, and 196 N applied load, 0.92 m/s sliding speed, and a total 3312 m sliding distance at ambient temperature in air.

The weights of the test samples and counterpart wheels were measured before and after the wear experiments using electronic scales with 0.1 mg accuracy. The volumetric wear loss, converted from the weight loss with the aid of a density measurement using the Archimedes principle, was employed to evaluate the wear resistance of test materials. The average friction coefficient μ was calculated according to the formula μ = *T*/*RP*, where *T* represents the friction torque, *R* represents the wheel radius, and *P* is the contact load applied on the block-like specimen. Worn surface morphologies, subsurface microstructure, and debris collected in the wear tests process of both Mo–40Ni–13Si alloy and reference test steels were finally observed by scanning electron microscope (SEM) and energy dispersive X-ray spectroscopy (EDS) analysis in order to explore the corresponding post-experimental wear mechanisms.

## 3. Results

### 3.1. Microstructure Characteristics

**Figure 2:**
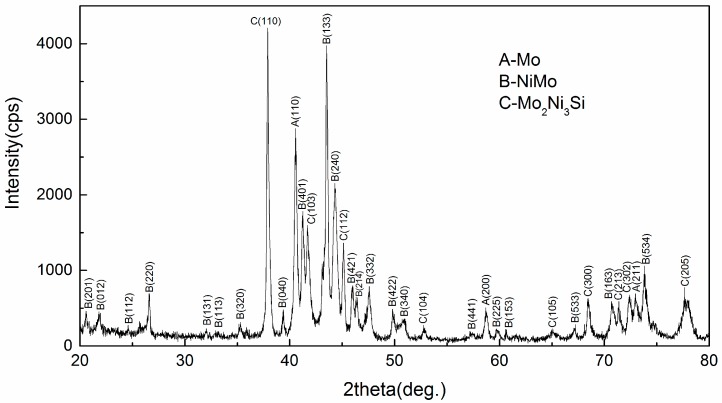
XRD profiles of the Mo–40Ni–13Si alloys.

As can be seen from XRD patterns shown in Figure 2, constituent phases of the Mo–40Ni–13Si alloy produced using in situ arc-melting route were binary intermetallic compound NiMo, ternary Mo_2_Ni_3_Si metal silicide with a topologically closed packed (TCP) phase having the *hP*12 MgZn_2_ type Laves crystal lattice, and refractory metal Mo phase. It is consistent with the expectation of alloy design.

**Figure 3:**
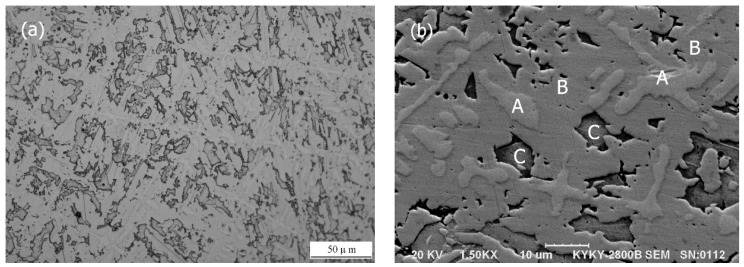
Low magnification optical microscope (OM) (**a**) and high magnification scanning electron microscope (SEM) (**b**) micrographs showing typical microstructure morphologies of the Mo–40Ni–13Si alloy.

Figure 3a shows the low magnification OM image of the typical microstructure morphologies of the Mo–40Ni–13Si alloy, which displays a uniform and dense microstructure. Three phases in the designed alloy could be identified clearly through careful examination of the SEM image with high magnification, as labeled in region A, B, and C in Figure 3b. Region A possesses the light gray dendritic morphology and was enriched in Mo by EDS analysis (seen in Table 1), while region B is the continuous gray matrix and its chemical composition was mainly Ni and Mo. Region C is a precipitation phase with an irregular shape and size, involving Ni, Mo, and Si three elements, as shown in Table 1.

Based on the XRD results and EDS analysis, the light gray dendritic phase dispersed uniformly in the microstructure was identified as the refractory metal Mo dissolved into a certain amount of Ni and Si, the continuous gray matrix is the binary intermetallic compound NiMo, while the irregularly shaped precipitation phase is ternary Mo_2_Ni_3_Si metal silicide. The volume fraction of refractory metal Mo dendrites and Mo_2_Ni_3_Si precipitation phase examined with the linear intercept method was about 6% and 34%, respectively.

### 3.2. Hardness and Density

The Mo–40Ni–13Si alloy is somewhat brittle for test bulk hardness due to being mainly composed of intermetallic phases, so the micro-hardness test was selected to give a hardness value. The alloy has a high hardness value about HV940, which is attributed to a high volume fraction of hard Mo_2_Ni_3_Si ternary metal silicide and NiMo intermetallic compound. The average hardness for individual Mo_2_Ni_3_Si and NiMo phases were HV1070 and HV910, respectively, carried on a HXZ-1000 micro-hardness indenter with a load of 20 g and a load dwell time of 15 s. It agrees satisfactorily with the results in NiMo/Mo_2_Ni_3_Si intermetallic composite [28]. Correspondingly, the indent size of the micro-hardness test ranges approximately from 5 to 7 μm for Mo_2_Ni_3_Si and from 6 to 8 μm for NiMo phase. It is difficult to accurately identify the hardness of ductile Mo phase because of its fine size in the Mo–40Ni–13Si alloy. In our previous work [29], the hardness value of Mo_SS_ in composites fabricated by laser melting and deposition rout was approximately HV620.

Density of the Mo–40Ni–13Si alloy is 9.17 g/cm^3^ detected with Archimedes’ principle. The density of reference hardened 0.45%C steel and austenitic stainless steel 1Cr18Ni9Ti is 7.81 g/cm^3^ and 7.79 g/cm^3^, identified using the same method for the purpose of comparing the wear volumetric loss.

### 3.3. Wear Resistant Property

**Figure 4:**
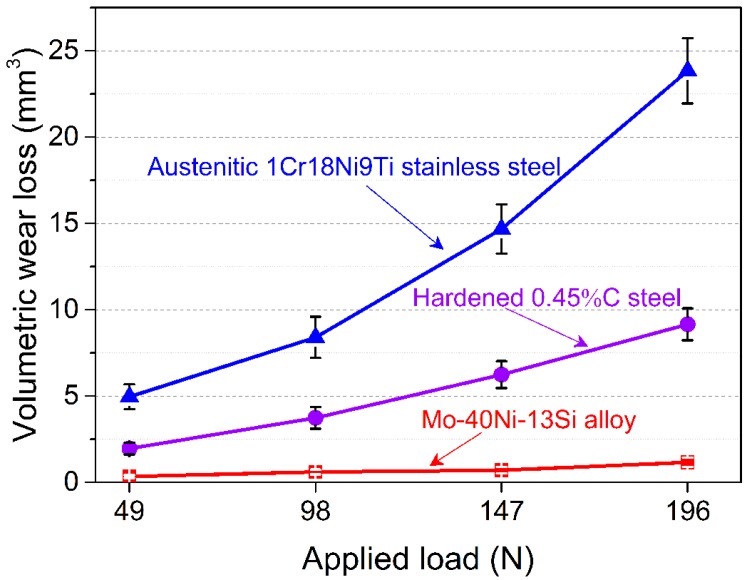
Volumetric wear loss of the Mo–40Ni–13Si alloys and reference test materials as a function of applied load under room temperature dry-sliding wear conditions.

The Mo–40Ni–13Si alloy exhibits outstanding wear resistant properties under room temperature dry sliding wear test conditions coupled with the hardened 1.0%C–1.5%Cr bearing steel mating wheel. Figure 4 shows the relationship between volumetric wear loss and the applied load of both the Mo–40Ni–13Si alloy and reference test materials. It can be seen that volumetric wear losses of the Mo–40Ni–13Si alloy are considerably lower than those of two comparison test materials under all selected wear test loads. Owing to the relative lower initial hardness, the wear resistance of austenitic 1Cr18Ni9Ti stainless steel is inferior to that of the hardened 0.45%C steel, which is reflected clearly in the wear test data at all contact loads.

Another phenomenon that needs to be noted is that the volumetric wear loss of the Mo–40Ni–13Si alloy increases quite slowly compared with the tremendous increase of the reference materials with the increase of applied load, as illustrated in Figure 4. The volumetric wear loss of austenitic 1Cr18Ni9Ti stainless steel is up to 20 times as high as that of the Mo–40Ni–13Si alloy when the contact load is 196 N. These results imply that the Mo–40Ni–13Si alloy has a lower wear-load coefficient than traditional engineering metallic materials at room temperature dry sliding wear conditions and thus is better when used under higher load wear environments.

### 3.4. Friction Coefficient

**Figure 5:**
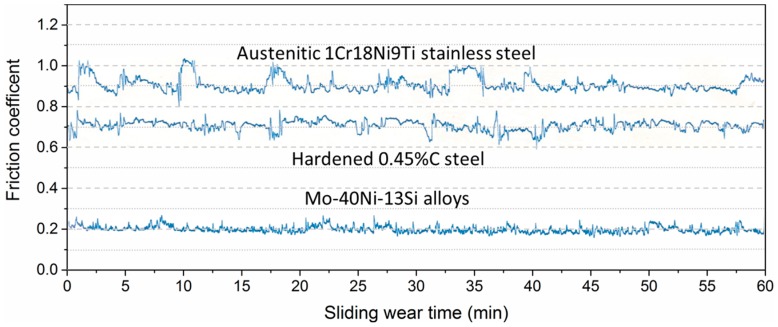
Friction coefficient profiles of the Mo–40Ni–13Si alloys and reference test materials as a function of wear time under 147 N applied load.

The friction coefficients along the sliding time were also continuously recorded during the wear tests. The friction traces of the Mo–40Ni–13Si alloy and two reference materials are clearly different under the contact load of 147 N, as given in Figure 5. The Mo–40Ni–13Si alloy shows low friction coefficients and a smooth friction trace (ranging from 0.14 to 0.26 and an average value of 0.2). The other curves of friction coefficient for two comparison materials manifest higher general values and large fluctuations throughout the test.

**Figure 6:**
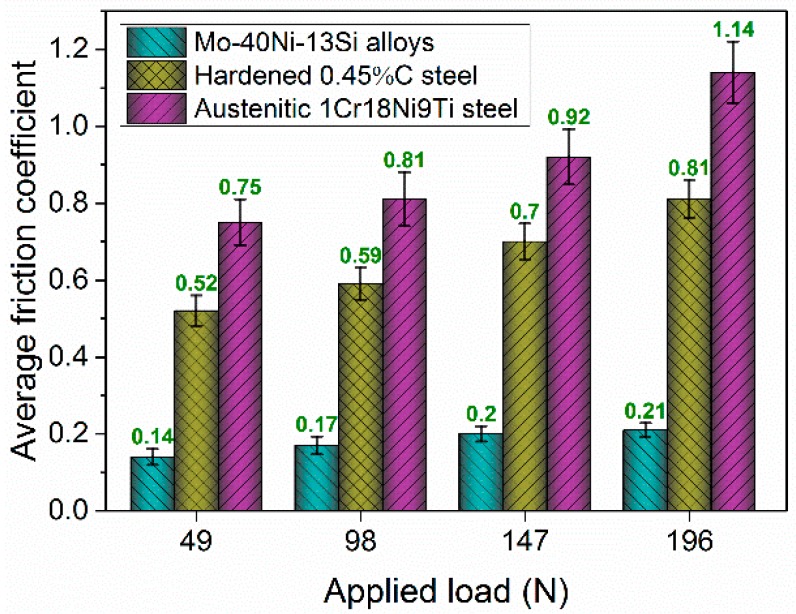
Average friction coefficient of the Mo–40Ni–13Si alloys and reference test materials at different applied loads.

In addition, as indicated in Figure 6, the average friction coefficient of the Mo–40Ni–13Si alloy is lower than the hardened 0.45%C steel and austenitic 1Cr18Ni9Ti stainless steel at any contact load, and is extremely insensitive to the contact load, while that of both comparison materials increase rapidly with the contact load increasing from 49 to 196 N. The possible explanation for the low friction coefficient of the Mo–40Ni–13Si alloy is that it has high hardness and outstanding adhesive and abrasive wear resistance resulting from the large volume fraction of intermetallic compound phases.

### 3.5. Metallic Tribological Compatibility

A good metallic material for tribological application should not only have prominent wear resistance properties and a low friction coefficient but also have a remarkable metallic tribological compatibility. Here, the metallic tribological compatibility refers to the tribo-metallurgical reciprocal compatibility, i.e., the degree of mutual solubility between metallic friction-pair materials.

A good metallic tribological compatibility means less solubility between the test alloy specimen and steel wheel. As shown in Table 2, the volumetric wear losses of either block-like samples or wear counterpart wheel (hardened 1.0%C–1.5%Cr steel) for the Mo–40Ni–13Si alloy is significantly lower than the two comparative materials at four selected loads. This result implies that the Mo–40Ni–13Si alloy has better metallic tribological compatibility compared to traditional engineering materials when coupled with a steel counterpart under room temperature dry sliding wear conditions. A rule of thumb in tribology is that the tribo-metallurgical reciprocal compatibility primarily relies on the atomic bonding type of wear contact counterparts. Hence, the covalent-dominant atomic bonding, which is different with the coupling bearing steel wheel, is responsible for the good metallic tribological compatibility.

### 3.6. Worn Surface Morphologies

**Figure 7:**
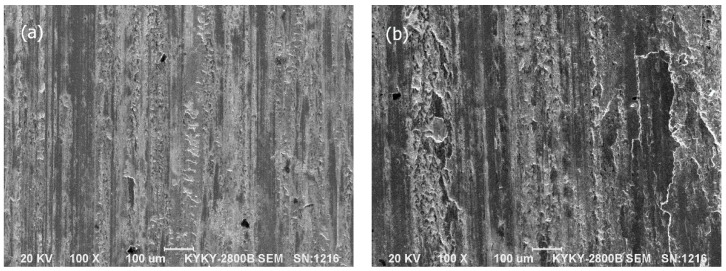
SEM micrographs showing the worn surface morphologies of the hardened 0.45%C steel (**a**) and austenitic 1Cr18Ni9Ti stainless steel (**b**) at a contact load of 196 N for a sliding distance of 3312 m.

**Figure 8:**
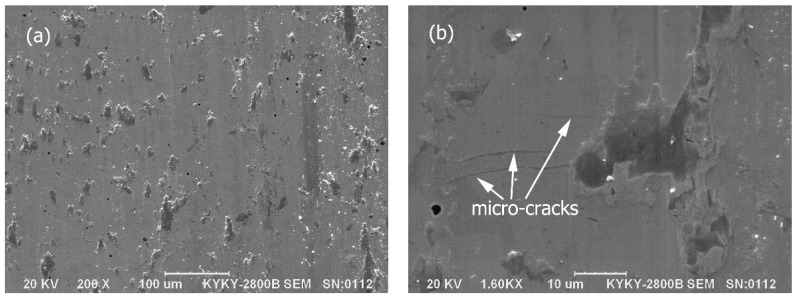
Low (**a**) and high (**b**) magnification SEM micrographs showing the worn surface morphologies of the Mo–40Ni–13Si alloy at a contact load of 196 N for a sliding distance of 3312 m.

**Figure 9:**
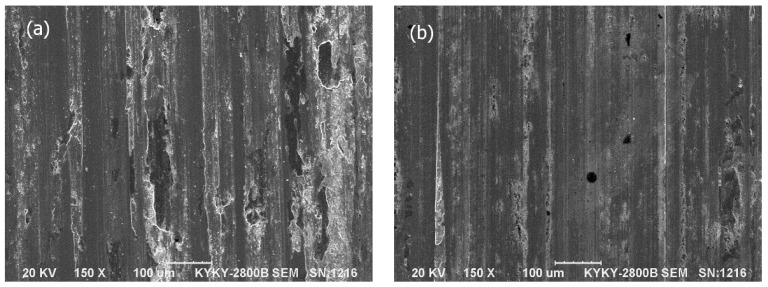

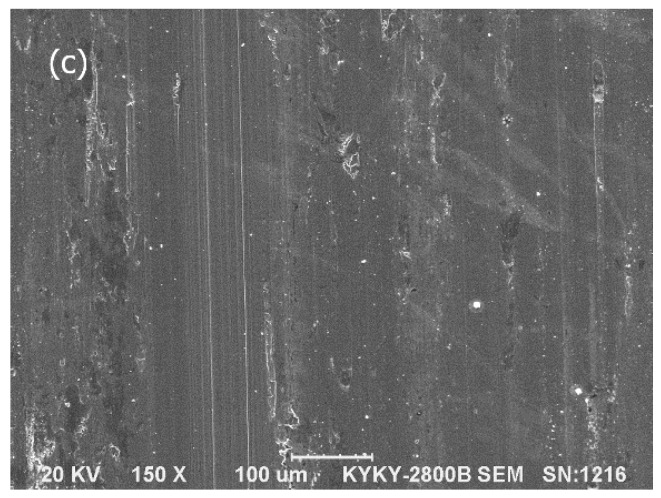
Worn surface morphologies of the hardened 1.0%C–1.5%Cr bearing steel wear counterpart wheel coupled with the hardened 0.45%C steel (**a**); austenitic 1Cr18Ni9Ti stainless steel (**b**); and the Mo–40Ni–13Si alloy (**c**), at a contact load of 196 N for a sliding distance of 3312 m.

The SEM micrographs of the worn surfaces of the Mo–40Ni–13Si alloy, two comparative materials (hardened 0.45%C steel and austenitic 1Cr18Ni9Ti stainless steel) and coupling 1.0%C–1.5%Cr bearing steel, tested at a contact load of 196 N and a sliding speed of 0.91 m/s for a total sliding distance of 3312 m, are given in Figure 7, Figure 8 and Figure 9.

It can be seen from Figure 7 that the worn surfaces of the hardened 0.45%C steel and austenitic 1Cr18Ni9Ti stainless steel are very rough and characterized by smearing, scratches, and plastic deformation. Smearing was more evident on the worn surfaces of austenitic 1Cr18Ni9Ti stainless steel, whereas the worn surface of hardened 0.45%C steel revealed more scratches. As for the rotating counterpart wheel, as shown in Figure 9a,b the worn surfaces coupled with both reference steels provide evidence of suffering adhesive and abrasive wear. There are more adhesive wear features on the worn surface coupling with 0.45%C steel (Figure 9a), but relatively less visible plowing compared to the coupling with austenitic stainless steel.

However, the worn surface of the Mo–40Ni–13Si alloy is quite smooth and clean, as indicated in Figure 8a, which is very consistent with the low friction coefficient and smooth trace mentioned above. There are no characteristic features of metallic adhesion and obvious abrasive wear—i.e., grooves and materials tearing. It should not be ignored that there exist a small amount of stuck wear debris particles and some island-like transferred cover layers on the worn surface of the Mo–40Ni–13Si alloy. The formation mechanism and protective contribution of transferred cover layers during the wear process has been discussed in our previous works [28].

Through careful examination of the high magnification SEM micrographs (Figure 8b) showing the worn surface, micro-cracks with the length from 5 to 30 μm were detected on the brittle intermetallic matrix. Interestingly, the propagation of micro-cracks stopped when approaching the ductile Mo phase distributed uniformly in microstructure of the Mo–40Ni–13Si alloy. Therefore, the intersection of micro-cracks and spalling of materials was avoided, which could be evidenced by the fact that no spalling fragments were found on the worn surface.

The worn surfaces of 1.0%C–1.5%Cr bearing steel rotating wheel coupling with the Mo–40Ni–13Si alloy displays extremely narrow micro-plowings and stuck tiny debris powders, as shown in Figure 9c, implying no adhesion between the block specimen and steel wheel during the sliding wear process. This phenomenon manifests once again that the Mo–40Ni–13Si alloy has less metallurgical solubility with traditional engineering steels and better tribological compatibility.

### 3.7. Wear Debris Morphology

**Figure 10:**
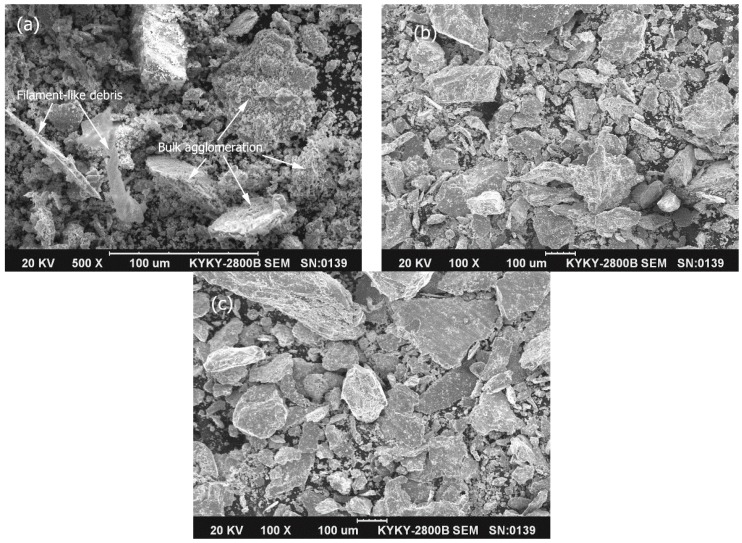
SEM micrographs of wear debris produced for the Mo–40Ni–13Si intermetallic alloy (**a**); the hardened 0.45%C steel (**b**); and austenitic 1Cr18Ni9Ti stainless steel (**c**).

Figure 10 shows the SEM micrographs of the wear debris collected at each end of wear test for three test materials. The difference in morphology of wear debris is consistent with the worn surface appearances, shown in Figure 7, Figure 8 and Figure 9.

For the designed Mo–40Ni–13Si intermetallic alloy, wear debris displays a size distribution from tiny powder, filament-like debris (up to 70 μm in length) to bulk agglomeration (up to 60 μm in size) at all applied loads, as illustrated in Figure 10a. Further EDS examination indicates that the chemical composition of the tiny powders is 55.44Fe11.01Ni7.93Mo4.06Si20.97O0.59Cr (at %). It is easy to deduce that the tiny wear debris powders predominantly originated from the wear counterpart wheel, hardened 1.0%C–1.5%Cr bearing steel. The filament-like debris is enriched Fe with measurable levels of Cr and O, which indicates that they are the cutting products from the steel wheel. Interestingly, the bulk of the debris is comprised of an agglomeration of tiny debris powders, which EDS results suggest are complex metal-based phases, with a certain amount of oxygen. In comparison, the wear debris of the hardened 0.45%C steel and austenitic 1Cr18Ni9Ti stainless steel are extremely large in size, as shown in Figure 10b,c.

### 3.8. Wear Subsurface

**Figure 11:**
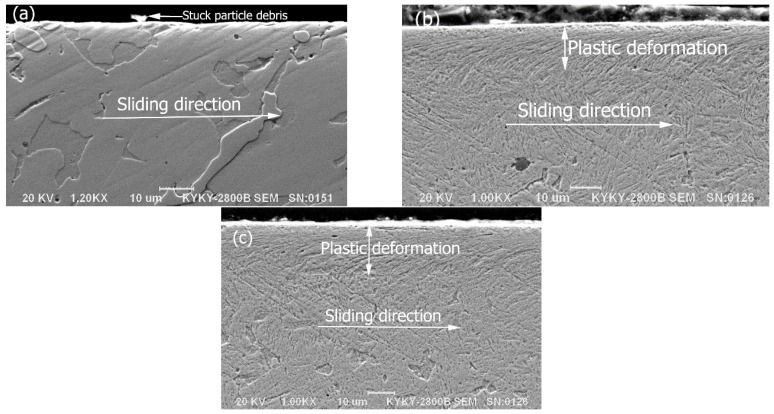
SEM micrographs showing subsurface morphologies of the Mo–40Ni–13Si intermetallic alloy (**a**); the hardened 0.45%C steel (**b**); and austenitic 1Cr18Ni9Ti stainless steel (**c**).

Figure 11 gives longitudinal cross-sections of the worn surfaces at a contact load of 196 N. It can be seen that no evidence of local plastic deformation, fracture, or selective wear on the wear subsurface were observed for the multiphase Mo–40Ni–13Si intermetallic alloys. In contrast, serious subsurface plastic deformation on the longitudinal section along the wear sliding direction occurred for the two comparing materials, hardened 0.45%C steel and austenitic 1Cr18Ni9Ti stainless steel. The two steels also exhibited similar depth of deformation under dry sliding wear conditions, which is probably owing to their similar hardness values.

Moreover, the Mo–40Ni–13Si alloy had similar morphologies of worn surface and subsurface at different loads. Thus, the wear resistance of the designed intermetallic alloy materials—including wear loss, surface wear, and subsurface morphologies—was independent on contact loads.

## 4. Discussion

The microstructure of the arc-melted Mo–40Ni–13Si intermetallic alloy (shown in Figure 3) demonstrates the presence of intermetallic NiMo phase, as well as a certain amount of molybdenum dendrites and ternary metal Mo_2_Ni_3_Si silicide at room temperature, which is consistent with the alloy design in high quality. Now, it is necessary to analyze the solidification process of the Mo–40Ni–13Si multiphase alloy because, up to the current knowledge accessible for the authors, little information is given concerning the solidification of the ternary Mo–Ni–Si system alloy.

Refractory metal Mo firstly started nucleating and growing as the liquid alloy cooled, because Mo has the highest melting point (2623 °C) among three phases of the Mo–40Ni–13Si alloy. In the very beginning of solidification, the nucleation location of the Mo crystalline grain is random in homogeneous melt if the heterogeneous nucleation on the surface of the crucible is not taken into account, which featured a uniform distribution of Mo dendrites in the microstructure. The Mo grain grew dendritically with successive solidifying and the change of solid and liquid phase composition depended on the local temperature and phase diagram.

Following that, the remaining liquid became relatively poor in Mo and rich in Ni, and the binary intermetallic compound NiMo phase formed surrounding Mo dendrites through peritectic transformation (L + α(Mo)→ δ(NiMo) at 1362 °C). As indicated in Table 1, an excessive amount of Si (approximately 10%) was dissolved in the NiMo phase, which considerably surpassed the limit in that Si in NiMo δ-phase should be less than 1% (at %) [30]. The reason behind this is possibly the incomplete diffusion of Si owing to non-equilibrium solidification of undercooled Mo–40Ni–13Si alloy melt at a fast cooling rates.

Ternary metal silicide Mo_2_Ni_3_Si is a typical *hP*12 MgZn_2_-type Laves phase with the lattice parameters of *a* = 0.47 nm and *c* = 0.747 nm. The MgZn_2_-type Laves phase was reported to be stable below 1200 °C [30]. Hence, further decreasing the melt temperature and continuously changing the element content in the molten alloy, Mo_2_Ni_3_Si phase formed as the solidification product of the remaining residual liquid in the last period of the solidification process.

The magnitude of the wear loss depends on the metallographic structure of the work-materials. In this regard, the novel Mo–40Ni–13Si multiphase intermetallic alloy appears to have an ideal microstructure. The refractory metal Mo is well known for its ductility and toughness, and the ternary metal silicide Mo_2_Ni_3_Si with topologically closed packed (TCP) structure possesses high hardness and strong atomic bonds. More importantly, the uniform distribution of ductile fine Mo dendritic phase and hard Mo_2_Ni_3_Si on continuous matrix composed by binary intermetallic compound NiMo (having a complex ordered orthorhombic pseudo-tetragonal structure [31]) provides the Mo–40Ni–13Si alloy an excellent combination of strength and toughness, which is undoubtedly beneficial for tribological properties.

Besides the microstructure, the wear of metallic materials is directly proportional to hardness. In resisting abrasive wear attacks, such as micro-cutting and -plowing, binary intermetallic compound NiMo and molybdenum nickel silicide Mo_2_Ni_3_Si phases play a critical role because of the inherent high hardness, under dry sliding wear test environments. Moreover, the Mo_2_Ni_3_Si phase scattered on continuous NiMo matrix can endure the frictional heating and maintain its high hardness during the dry sliding wear process even under a high contact load of 196 N, which resulted from the anomalous hardness-temperature relationship of metal silicides.

The behavior of metallic materials in wear conditions is not determined by microstructure and hardness, but also by chemical affinity of decoupled wear elements. The two steel comparison materials have the same metallic bond with the wear coupling wheel, which lead to a strong chemical affinity and serious wear loss. While the covalent-dominant atomic bonding attributes of NiMo and Mo_2_Ni_3_Si phases in the Mo–40Ni–13Si alloy lead to poor chemical affinity with its wear steel counterpart. It is the reason why no metallic adhesion occurred between the sliding surfaces when the novel intermetallic alloy had a slide-interaction with the metallic counterpart (hardened 1.0%C–1.5%Cr bearing steel wheel). Therefore, the strong covalent-dominant atomic bonding attributes of NiMo and Mo_2_Ni_3_Si phases endow the Mo–40Ni–13Si alloy excellent resistant capability to adhesive wear damages from metallic bonding to its steel counterpart, and prevented the intermetallic matrix from plastic deformation, adhesion, and materials-transferring, as well as a welding joint to the metallic asperities on the contact surface of the slide-coupling metallic counterpart. Both the block-like alloy sample and metallic coupling wheel gave a relatively smooth worn surface after the wear test, as indicated in Figure 8 and Figure 9c. Furthermore, the low friction coefficient and excellent metallic tribological compatibility of the Mo–40Ni–13Si alloy are also apparently results of the different atomic bonds with the coupling steel wheel

Besides the above mentioned, the increase in wear resistance of the Mo–40Ni–13Si alloy can also be associated with the contribution of ductile Mo phase on toughness improvement. Note that some micro-cracks formed in the brittle intermetallic compound matrix of the Mo–40Ni–13Si alloy, as displayed in Figure 8b. In the friction and wear test process, the block-like alloy sample suffered not only normal compressive stress from the coupling steel wheel but also shear stress induced by friction between two contact surfaces, which is responsible for the formation of micro-cracks on the brittle intermetallic matrix. The driving force to initiate micro-crack extending is higher relative to NiMo and Mo_2_Ni_3_Si phase when the micro-cracks grew approaching the ductile Mo phase. The propagating crack became locally impeded in front of ductile Mo dendrites. These phenomena imply that the in situ incorporation and even distribution of refractory metal Mo with high ductility and toughness in the intermetallic compound matrix also had a dominant role in supplying an excellent toughening effect. The increase in ductility is also a highly desirable property for wear resistance intermetallic-based materials. Combining the worn surface and debris morphologies, taking the friction coefficient into account, the dry sliding wear process could be deduced as follows. For two reference metallic materials, serious abrasive and adhesive wear practices, which are characterized by unwanted displacement and attachment of wear debris and materials from one surface to another, happened following the short early run-in period. Whereas, for the novel Mo–40Ni–13Si alloy, the adhesion on contact surfaces is difficult owing to the different atomic bonding features between the test alloy and coupling 1.0%C–1.5%Cr bearing steel wheel. The rotating steel wheel was removed gradually in the form of micro-cutting by coupling the block-like alloy specimen. The metal debris, mainly produced from the steel wheel, was oxidized under the effect of friction heat. Some wear debris on the sliding surfaces became metal oxide layers during the sliding motion, which produced an anti-wear effect. The tiny debris particles likely act as a good solid lubricant between the wear couples and hence are helpful to the improving of wear resistance and metallic tribological compatibility. Therefore, the Mo–40Ni–13Si alloy only suffered soft abrasive wear from debris and superficial oxidation due to tribo-chemical reactions. This is highly consistent with the low wear loss and smooth worn surface.

## 5. Conclusions

A novel wear resistant Mo–40Ni–13Si multi-phase intermetallic alloy was fabricated successfully with Mo–Ni–Si powder blends by arc-melting technique. The microstructure of the Mo–40Ni–13Si alloy shows a uniform distribution of primary refractory metal Mo dendrites on continuous binary intermetallic compound NiMo matrix as well as a certain amount of ternary metal silicide Mo_2_Ni_3_Si precipitation phase. The Mo–40Ni–13Si alloy exhibits outstanding wear resistant property and sluggish wear-load characteristics under selected room temperature dry sliding wear conditions, which are attributed to its unique microstructural features and effective combination of strength, ductility, and toughness. Ductile Mo dendritic phase plays a positive role in toughening and improving wear resistance of the intermetallic alloy through stopping the propagation of micro-cracks over the duration of wear tests. The dominant mechanism of material removal and wear for the Mo–40Ni–13Si alloy is soft abrasive wear in a room temperature, dry sliding wear environment.

## Figures and Tables

**Table 1:** EDS results of individual phase in the Mo–40Ni–13Si alloy.

Phase (Region in Figure 3b)	Content of Element (at %)		
	Mo	Ni	Si
Light gray dendrite (A)	93.19	4.45	2.36
Continuous gray matrix (B)	42.82	46.73	10.45
Precipitation phase on continuous matrix (C)	30.93	53.01	16.06

**Table 2:** Volumetric wear loss of block samples and coupling steel wheels under dry sliding wear conditions (mm^3^).

Load (N)	Mo–40Ni–13Si Alloys	Coupling Wheel	Hardened 0.45%C Steel	Coupling Wheel	Austenitic 1Cr18Ni9Ti Stainless Steel	Coupling Wheel
49	0.34	2.08	1.96	7.11	4.96	11.76
98	0.60	3.57	3.74	10.28	8.40	21.14
147	0.69	5.92	6.24	23.63	14.67	36.71
196	1.16	7.35	9.15	37.42	23.84	58.06
